# Hollow Graphitic Nanoshells as a Material for Ion Batteries

**DOI:** 10.3390/ma19061187

**Published:** 2026-03-18

**Authors:** Maria Hasan, Alicja Bachmatiuk, Gražyna Simha Martynková, Karla Čech Barabaszová, Mark H. Rümmeli

**Affiliations:** 1 Electron Beam Emergent Additive Manufacturing (EBEAM) Centre, Centre for Nanotechnlogy (CNT), Centre for Energy and Environmental Technologies (CEET), VSB-Technical University of Ostrava, 17. Listopadu 15, 70800 Ostrava, Czech Republicalicja.bachmatiuk@vsb.cz (A.B.); 2 Centre for Nanotechnology (CNT), Centre for Energy and Environmental Technology (CEET), VSB-Technical University of Ostrava, 70800 Ostrava, Czech Republic; grazyna.simha@vsb.cz (G.S.M.); karla.cech.barabaszova@vsb.cz (K.Č.B.); 3 Faculty of Chemistry, Wroclaw University of Science and Technology, Wybrzeże Wyspiańskiego 27, 50-370 Wroclaw, Poland; 4 Key Laboratory of Advanced Carbon Materials and Wearable Energy Technologies of Jiangsu Province, Key Laboratory of Core Technology of High Specific Energy Battery and Key Materials for Petroleum and Chemical Industry, Soochow Institute for Energy and Materials Innovation, College of Energy, Soochow University, Suzhou 215006, China; 5 Institute for Materials Chemistry, IFW Dresden, 20 Helmholtz Strasse, 01069 Dresden, Germany

**Keywords:** hollow structures, hollow cages, rechargeable batteries, graphitic structures, carbonaceous structures

## Abstract

Hollow graphitic nanoshells (HGSs) are widely investigated as battery materials because their conductive shells and internal voids can simultaneously influence ion transport, electron percolation, and mechanical stress accommodation. Yet, the field remains largely morphology-driven, with performance often attributed generically to “hollowness” rather than to structural parameters. This review examines HGSs from a parameter-oriented perspective. It highlights key structural features, including graphitization degree, shell thickness, cavity size, pore architecture, and defect or dopant chemistry. These features collectively shape electrochemical behavior. We discuss how these features influence transport kinetics, interphase stability, volumetric efficiency, and mechanical resilience across insertion, metal anode, multivalent, solid-state, and halogen chemistries. Major synthesis approaches, including hard-templated, soft-templated, self-templated, and biomass-derived routes, are evaluated based on the structural control they provide and the influence of synthesis conditions on shell architecture, graphitic ordering, and pore structure. Special attention is given to how these structural features develop during processing and how they affect ion accessibility, conductivity, and stability. Finally, we outline a shift toward quantitative, parameter-driven engineering supported by operando diagnostics, electrode-level modeling, and standardized reporting. HGSs will only achieve practical relevance when structural optimization extends beyond particle morphology to transport uniformity, interfacial stability, network connectivity, and life-cycle responsibility.

## 1. Introduction

The rapid growth of electrochemical energy storage technologies has intensified the search for carbon architectures that combine high electrical conductivity, structural robustness, and tunable ion storage behavior. Hollow carbon nanostructures, particularly hollow graphitic nanoshells (HGSs), have emerged as a distinct material class because they integrate a conductive sp^2^ carbon shell with an internal void space capable of accommodating mechanical stress and facilitating ion transport. Comprehensive reviews on hollow carbon nanocages and template-assisted porous carbons document the evolution of this field and its relevance to battery systems [1,2].

Despite extensive reports of improved electrochemical performance, the benefits of HGSs are often attributed generically to “hollowness,” without clearly isolating which structural parameters govern transport kinetics, interphase stability, or mechanical resilience. In practice, hollow morphology alone does not guarantee superior battery behavior. Excessive surface area can intensify electrolyte decomposition and reduce initial Coulombic efficiency in porous carbon anodes [3,4]. Highly ordered graphitic shells enhance electronic conductivity but may restrict ion accessibility, particularly for larger ions such as Na^+^ and K^+^, where interlayer spacing and defect chemistry become decisive [3]. Conversely, excessive structural disorder improves adsorption-based storage yet promotes parasitic reactions and unstable solid–electrolyte interphases [5]. These trade-offs indicate that electrochemical performance is not an intrinsic consequence of hollow geometry but rather of the quantitative balance among structural variables.

A rigorous interpretation therefore treats HGSs as a coupled parameter system defined by graphitization degree, shell thickness, cavity size, pore size distribution, and defect or heteroatom chemistry. Graphitic ordering governs electronic percolation and insertion thermodynamics, while shell thickness sets diffusion length and mechanical strength. The cavity buffers stress but penalizes volumetric energy density when oversized, an issue increasingly emphasized in practical electrode engineering [6]. Hierarchical porosity regulates electrolyte infiltration and ion transport yet must be controlled to prevent excessive interfacial reactivity [7]. Defect engineering and heteroatom doping modify local charge density and ion affinity, but excessive disruption of the sp^2^ network increases charge transfer resistance and long-term instability [8,9]. Because these parameters evolve simultaneously during synthesis and thermal treatment, optimizing one inevitably perturbs the others.

Although substantial progress has been made, the field remains largely morphology-driven, with limited quantitative mapping between shell geometry, pore architecture, defect density, and measurable transport metrics. Recent hard carbon design analyses emphasize the need for predictive structure–performance relationships and standardized reporting beyond gravimetric capacity alone [3]. For HGSs to transition from academic demonstrations to technologically relevant battery components, structural optimization must extend beyond hollow morphology toward parameter-driven engineering constrained by transport uniformity, volumetric efficiency, interfacial stability, electrode-level connectivity, and life-cycle sustainability.

**Figure 1:**
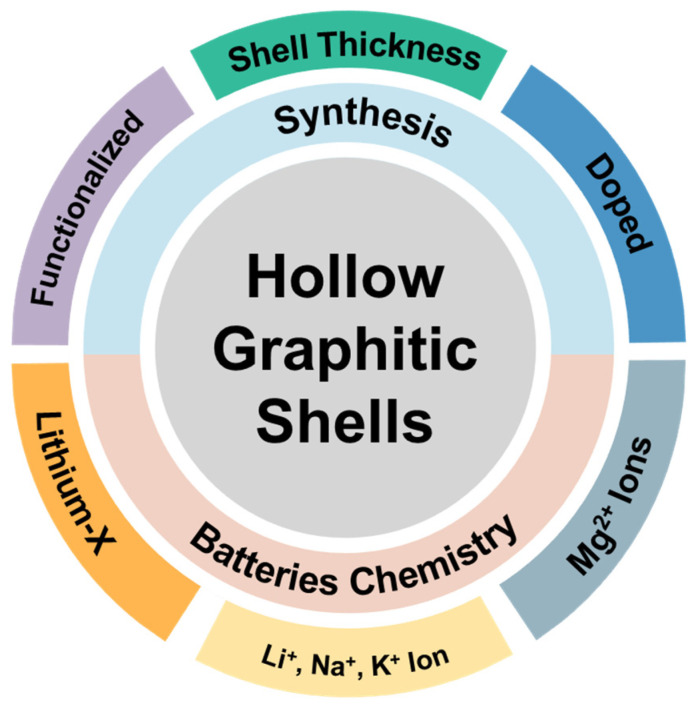
Schematic representation of key aspects covered in this review.

To meaningfully connect nanoscale architecture with practical performance, this review first examines structural design principles for HGS, because understanding how parameters such as graphitic order, shell thickness, porosity, and cavity geometry influence ion transport, interfacial stability, and mechanical behavior is essential for rational material engineering rather than descriptive morphology alone. We then discuss the major preparation strategies (hard-templated, soft-templated, self-templated, and biomass-derived approaches), selected because they represent the dominant and practically relevant pathways for controlling shell geometry, pore architecture, graphitic continuity, and dopant integration while spanning different levels of precision, scalability, and environmental burden. Finally, we review battery chemistries across alkali-ion, multivalent, and solid-state systems, as these leading rechargeable technologies impose distinct electrochemical constraints that demand chemistry-specific structural tuning. We further extend the discussion beyond performance metrics to address environmental impact, manufacturing intensity, unresolved structural limitations, and the need for quantitative, parameter-driven engineering frameworks, positioning HGSs within a broader context of sustainable and industrially viable energy storage. Figure 1 schematically summarizes HGSs as a parameter-defined materials platform.

## 2. Structural Designs and Engineering in HGS

Electrochemical performance in a single HGS does not arise from hollowness alone. Rather, it emerges from the coupled optimization of graphitic ordering, shell thickness, cavity size, pore architecture, and defect/dopant chemistry. These parameters are interdependent: modifying one typically alters several others during synthesis or thermal treatment. Therefore, understanding HGSs requires moving beyond morphology description toward identifying how each structural variable governs transport, reactivity, and mechanical stability. In the following sections, these structural parameters are discussed individually in detail, with emphasis on how each governs the electrochemical behavior of HGSs and their resulting performance in battery systems.

### 2.1. Graphitization Degree

The degree of graphitization determines the balance between electronic conductivity and ion storage activity. Increasing sp^2^ content enhances electronic transport, improves rate capability, and stabilizes the electrode–electrolyte interface by forming continuous conductive pathways. Highly ordered graphitic domains also suppress excessive parasitic reactions during cycling [10].

However, excessive ordering reduces electrochemical activity. As graphitization increases, defect density and pore accessibility decrease, limiting surface-mediated storage and ion transport pathways. In highly crystalline graphite-like shells, ion storage becomes dominated by intercalation with restricted diffusion channels, leading to increased polarization, particularly for larger ions such as Na^+^ or K^+^. Conversely, insufficient graphitization introduces excessive structural disorder and sp^3^-rich regions, resulting in poor electronic conductivity, unstable interphases, and strong ion trapping at defects, which lowers initial Coulombic efficiency [11]. Moderately graphitized turbostratic shells provide the most effective compromise. Expanded interlayer spacing facilitates ion insertion while maintaining sufficient conductive continuity, reducing diffusion barriers for larger ions such as Na^+^ [12]. Thus, optimal performance is achieved not at maximum crystallinity but at intermediate graphitic ordering that preserves both transport and storage functionality.

### 2.2. Shell Thickness

Shell thickness defines both ion diffusion distance and structural robustness in HGS, directly influencing charge transfer resistance and overall electrochemical performance [13,14,15]. Because ions must traverse the carbon shell to access both external and internal storage sites, the shell functions as a primary transport barrier [13,16]. Thinner shells reduce diffusion resistance and enhance reaction kinetics, leading to improved rate capability, particularly under high-current operation [14,15,17].

However, thinner is not universally better. Ultrathin shells, especially those formed under incomplete or non-uniform carbonization, often exhibit reduced mechanical stability and structural discontinuities, making them prone to deformation or fracture during repeated cycling [13]. In contrast, thicker shells provide greater resistance to electrochemical stress and improved structural integrity but at the expense of longer ion transport pathways and reduced effective utilization of the internal cavity [15,16]. Although intermediate shell thicknesses are often reported to deliver optimal performance, ultrathin shells in the range of ~10–15 nm can remain stable and achieve exceptional rate capability when the carbon framework is sufficiently continuous and well carbonized [14,18]. Therefore, optimal shell thickness represents a system-specific balance between kinetic accessibility and mechanical resilience rather than a universal minimum value [15,16].

### 2.3. Cavity Size

The internal cavity is a functional design parameter rather than merely a geometric feature. It buffers volume expansion of active phases, mitigates shell fracture, and preserves electrical contact during repeated cycling, thereby enhancing structural stability and capacity retention compared to solid counterparts [19,20]. However, increasing cavity size is not universally beneficial. Excess void volume reduces volumetric energy density and packing efficiency, lowering tap density and potentially limiting practical electrode performance despite favorable gravimetric capacity [19]. Cavity dimensions are inherently coupled to shell thickness and carbon quality. During carbonization, shrinkage and densification alter void size, and poorly ordered or weakly graphitized shells may partially collapse, reducing effective cavity volume [13,19]. In template-assisted systems, cavity size can be predictably tuned through template selection and shell growth conditions, whereas in template-free routes, it depends on internal phase separation and mass loss dynamics during pyrolysis [13,21]. Therefore, cavity size must be engineered in proportion to the expected active material expansion or deposition volume. Oversizing cavities without regard to structural integrity and volumetric penalties does not guarantee improved battery performance [19,21].

### 2.4. Porosity and Pore Size Distribution

Porosity governs electrolyte accessibility, ion transport pathways, and effective active material utilization in HGSs [7,14,15]. Mesopores enable rapid electrolyte infiltration and reduce diffusion limitations, thereby improving rate capability and high-power performance, while hollow carbon frameworks can further shorten ion transport pathways during electrochemical reactions [7,15,17].

Macroporous interparticle networks facilitate electrolyte transport in thicker electrodes and help accommodate strain during cycling, contributing to structural stability [7,22]. Micropores and defect-associated nanovoids increase surface area and provide additional adsorption-based storage sites. However, excessive microporosity promotes electrolyte decomposition and irreversible capacity loss during initial cycling, particularly in highly disordered carbons [14,15].

Poorly controlled pore structures can accelerate interfacial side reactions and destabilize the solid–electrolyte interphase, leading to performance decay [14,15]. Thus, maximizing surface area alone is not a reliable design strategy. Effective HGS architectures prioritize mesopore-dominated hierarchies with controlled microporosity, ensuring rapid ion transport while limiting excessive interfacial reactivity [7,15].

### 2.5. Defect Engineering and Heteroatom Doping

Defects and heteroatom dopants introduce chemically active sites and modify the electronic structure of HGS. Vacancies, edge sites, and substitutional dopants create additional ion adsorption and insertion locations, altering intercalation energetics and increasing reversible capacity [8,23]. These defect sites also serve as entry points that facilitate ion transport across the carbon framework, effectively lowering diffusion barriers and enhancing reaction kinetics [24].

**Figure 2:**
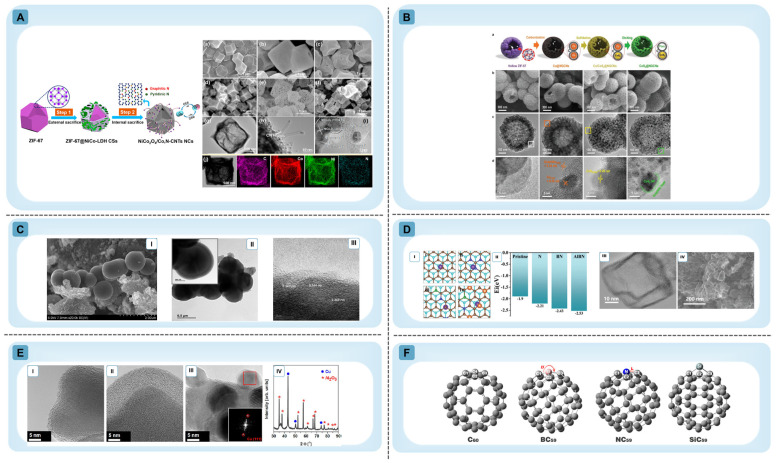
Representative strategies and structural characterization of heteroatom-doped HGSs illustrating how defect engineering and dopant incorporation modify carbon architectures and electronic properties. (**A**) Schematic synthesis of NiCo_2_O_4_/Co,N-CNTs NCs via a self-sacrifice template strategy. FE-SEM (**a**–**f**), TEM (**g**,**h**), HRTEM (**i**), and elemental mapping ((**j**); C, Co, Ni, N). Reprinted with permission from Ref. [26] Copyright Copyright 2018 American Chemical Society. (**B**) (**a**) Synthesis scheme of mesoporous hollow CoS_2_@NGCNs (inset: shows an image of a brain coral). (**b**) SEM, (**c**) TEM, and (**d**) HRTEM images of ZIF-67 and derived Co-based hollow NGCNs (left to right). Reprinted with permission from Ref. [27] copyright 2019 WILEY-VCH Verlag GmbH & Co. KGaA, Weinheim. (**C**) Morphology and graphitic lattice structure of P-doped HGS. (**I**) FESEM image, (**II**,**III**) HRTEM image Reprinted with permission from Ref. [28] Copyright 2021 the authors Springer Nature. (**D**) Morphology and expanded interlayer structure of AlBN-CNCs. DFT simulation (**I**) Li intercalation configurations on (**i**) pristine graphene, (**ii**) N-doped graphene, (**iii**) B–N-doped graphene, and (**iv**) Al–B–N-doped graphene. (**II**) Intercalation energy diagrams of Li ion on different atoms doped graphene configurations. (**III**) SEM image (**IV**) TEM image of AlBN-CNC. Reprinted with permission from Ref. [8] Copyright 2024 small, Wiley-VCH GmbH. (**E**) TEM images of graphene-coated aluminum oxide nanoparticles: (**I**) starting material, (**II**) material functionalized with thiol groups, (**III**) material decorated with copper particles, (**IV**) XRD spectrum of the material decorated with copper particles. Reprinted with permission from Ref. [34] Copyright 2013 American Chemical Society. (**F**) Optimized geometries of pristine and heteroatom-doped C_60_ (B, N, Si). Reprinted with permission from Ref. [29] Copyright 2025, the author(s).

Controlled dopant incorporation, such as that achieved through MOF-derived carbonization strategies (Figure 2A) [25,26], enables homogeneous distribution of heteroatoms and metal species within graphitic shells (Figure 2B) [27]. Heteroatom incorporation (e.g., N, P, B) modulates local charge density and can expand interlayer spacing, thereby improving ion transport kinetics while tuning the electronic structure [8,28]. Representative systems such as P-doped and La/N co-doped HGSs (Figure 2C,D) further demonstrate how tailored bonding environments and electronic polarization can be introduced without disrupting the overall graphitic framework. Density functional theory and experimental validation confirm that dopant-induced charge redistribution lowers ion intercalation energies while preserving conductive continuity (Figure 2E) [8]. At the atomic scale, substitutional heteroatoms induce localized strain and bond polarization in curved carbon architectures (Figure 2F), highlighting the intrinsic structural consequences of doping in sp^2^ carbon systems [29].

Beyond transport modulation, certain dopants, particularly phosphorus, also regulate interphase chemistry by promoting thinner and more stable solid–electrolyte interphases, thereby improving initial Coulombic efficiency and long-term cycling stability [30].

However, excessive defect density is detrimental. High structural disorder disrupts sp^2^ continuity, reduces electronic conductivity, and increases charge transfer resistance, leading to greater polarization during cycling [31]. Moreover, highly reactive defect-rich surfaces accelerate electrolyte decomposition and excessive SEI formation, resulting in substantial irreversible capacity loss during early cycles [32]. Thus, defect engineering must be carefully controlled. The objective is to introduce chemically active sites that enhance ion storage and kinetics while preserving a continuous conductive backbone. When properly optimized, heteroatom doping improves reversible capacity, rate capability, and interfacial stability without compromising structural coherence or long-term electrochemical durability [29,33].

## 3. Preparation Methods of Hollow Carbon Nanocages

The application-oriented performance of HGSs in battery systems originates not simply from their hollow interior but from the coordinated control of shell thickness, cavity size, graphitic ordering, pore architecture, and defect chemistry [2,35,36]. The hollow cavity governs volumetric buffering and internal deposition space, whereas the graphitic shell mediates ion transport, electron conduction, and interfacial stability. Consequently, synthesis routes must be evaluated according to the structural parameters they control and the precision with which they do so. In this context, hard-templated, soft-templated, and self-templated strategies represent different structural control frameworks rather than merely alternative preparation procedures.

### 3.1. Hard-Template Methods

Hard-template methods are the most reliable way to produce well-defined HGSs because the sacrificial template controls the cavity size, shell shape, and overall structure before carbonization, while precursor deposition and heat treatment determine the shell thickness and continuity. However, their practical significance extends beyond morphology control. It also depends on how well they balance structural precision with graphitization control, scalability, and environmental impact. As shown schematically in Figure 1, the template transfers its geometry to the material, allowing predictable control of internal void size and pore connectivity. This directly affects ion diffusion and electrolyte accessibility [2,35,36]. However, recent evidence shows that geometry inheritance alone is not enough. Increasing attention is now given to “process inheritance,” where the template not only defines the shape but also influences carbon ordering, dopant incorporation, and defect formation during thermal treatment. From this perspective, the key factor is the ability to control the shell microstructure under scalable processing conditions, rather than simply maintaining cavity shape [2,37]. Spherical templates are particularly attractive for battery electrodes because they enable uniform stress distribution, high packing density, and strong mechanical stability during cycling. In contrast, polyhedral shapes can create uneven curvature and localized surface reactivity [1,20]. However, in real electrodes with practical thickness, performance is influenced not only by particle shape but also by how particles interact within the electrode. Electronic conduction depends on the percolation network formed by conductive additives, while ionic transport is controlled by the relationship between porosity and tortuosity as the electrode becomes thicker [38].

Modeling studies show that lithium diffusion through particle agglomerates can create greater resistance than diffusion within individual particles. This means that improving the hollow structure of single particles does not always lead to better overall electrode performance [39].

Operando X-ray diffraction and tomography studies also reveal uneven lithiation and particle fracture in thick electrodes, especially near the separator. These observations indicate that electrochemical gradients cause mechanical and reaction inhomogeneities that cannot be predicted from single-particle symmetry alone [38,40].

**Figure 3:**
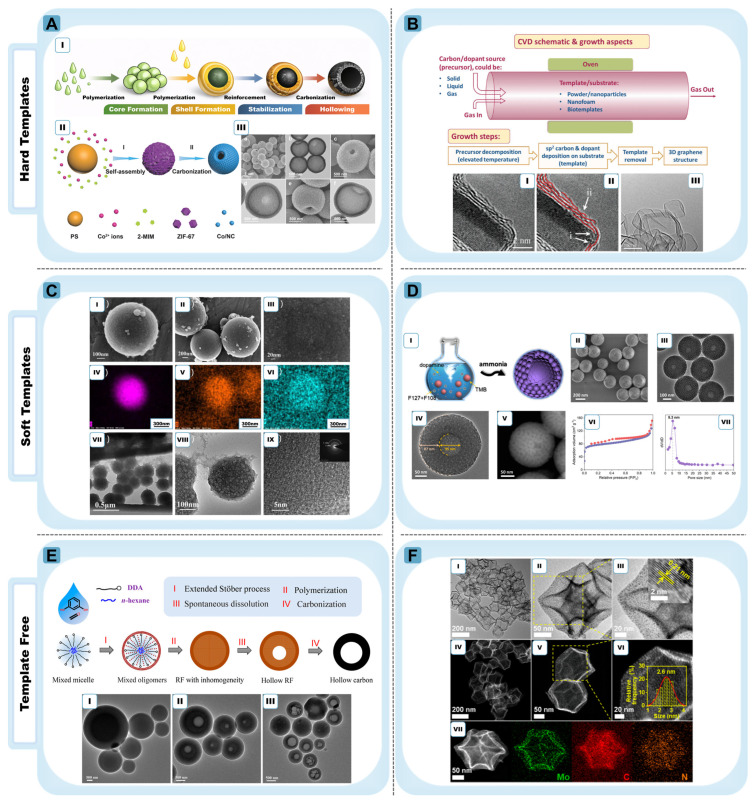
Representative synthesis strategies and structural characterization of HGSs: (**A**) Hard-template synthesis of HGSs (**I**) Preparation of HGSs via carbonization of core-shell polymer particles. (**II**) Controlled formation of PS@ZIF-67 composite particles followed by thermal conversion into single-holed Co/N-doped carbon (Co/NC) hollow structures. (**III**,**a**–**f**) Corresponding FESEM and TEM images confirming hollow morphology. Reprinted with permission from Ref. [43] © 2017 The Authors. Published by WILEY-VCH Verlag GmbH & Co. KGaA, Weinheim (**B**) General presentation of Template-assisted thermal CVD approach. Precursors (solid, liquid, or gas) are introduced into a hot CVD reactor, where controlled parameters (temperature, time, pressure, flow rate, carrier gas) enable decomposition and deposition of sp^2^ carbon and dopants (e.g., N) onto 3D/porous templates (e.g., Ni foam, MgO nanoparticles). Subsequent template etching yields high-quality (doped) 3D graphene architectures. Reproduced with permission from [44]. Copyright 2019 WILEY-VCH Verlag GmbH & Co. KGaA, Weinheim. TEM images. (**I**–**III**) show (**I**) graphene-coated MgO crystals, (**II**) highlighted graphene layers, wrinkle formation during growth, and (**III**) encapsulated graphitic structures after MgO removal. Reprinted with permission from Ref. [45]. Copyright 2007 American Chemical Society. (**C**) Structural and compositional characterization of PMCs-2: (**I**–**III**) SEM images, (**IV**–**VI**) EDS elemental mapping, (**VII**–**IX**) TEM images demonstrating morphology and homogeneous elemental distribution. Reprinted with permission from Ref. [49] Copyright 2025 Springer Nature. (**D**) Synthesis and characterization of ordered mesoporous HGSs: (**I**) Schematic illustration of the synthesis process of mesoporous HGS; FESEM image (**II**), TEM image (**III**,**IV**), and HAADF-STEM (**V**) images confirming the hollow and ordered mesoporous structure; nitrogen adsorption–desorption isotherms (**VI**) and pore size distribution (**VII**) after calcination. Reprinted with permission from Ref. [50] Copyright 2024 American Chemical Society. (**E**) Template-free formation of hollow resorcinol–formaldehyde (RF) spheres via extended Stöber process, spontaneous internal dissolution, and carbonization. (**I**) TEM images show RF spheres, (**II**) hollow RF after ethanol etching, and (**III**) hollow carbon spheres after carbonization under N_2_ at 550 °C. Reprinted with permission from Ref. [51] Copyright 2024 American Chemical Society (**F**) MoC/N-doped carbon nanocomposites (MoC/NCNCs). TEM images (**I**–**III**) reveal hollow structures with embedded MoC nanoparticles; the 0.21 nm lattice spacing corresponds to the MoC (200) plane. STEM images (**IV**–**VI**) and size distribution analysis confirm nanoparticle dispersion, while elemental mapping (**VII**) shows uniform Mo, C, and N distribution. Reprinted with permission from Ref. [52] Copyright 2022 American Chemical Society.

The hierarchical pore architecture typically associated with hard-templated HGSs consists of micropores and mesopores within the shell and a central hollow cavity (Figure 3). Micropores commonly arise from polymer decomposition and framework contraction during carbonization, whereas mesoporosity may originate from template texture or pore-directing agents [2,35,36]. While such multiscale porosity promotes rapid ion diffusion and mass transport, excessive microporosity may increase surface reactivity and compromise initial Coulombic efficiency. Therefore, porosity must be balanced against shell continuity and graphitic order. Recent studies have begun to explore strategies that partially decouple graphitic ordering from defect density by introducing spatial heterogeneity within carbon architectures. In such designs, regions of relatively higher graphitic order can facilitate electron transport, while more disordered or defect-rich domains preserve ion-accessible storage sites. These structurally heterogeneous shells suggest that the conventional graphitization–defect trade-off may be mitigated through controlled microstructural gradients rather than treated as a strictly binary compromise [41].

From a structural perspective, polymer spheres enable precise cavity formation because their uniform size and smooth surfaces allow conformal precursor coating. In core–shell polymer systems, the hollow architecture is inherited directly from the precursor structure: the core diameter determines the internal cavity size, while the shell thickness governs the thickness of the resulting carbon wall after carbonization (as shown in Figure 3A(I)). This relationship is demonstrated in PMMA/PAN core–shell particles prepared through two-stage polymerization, where PMMA spheres (~200 nm) are coated with a PAN shell (~30 nm) that subsequently converts into a carbon framework during heat treatment, producing hollow carbon nanospheres with cavities of approximately 100–150 nm and carbon shells of ~15 nm [42].

During thermal treatment, the hollow structure forms through selective decomposition of the core polymer, while the shell converts into carbon. In the PMMA/PAN system, the PMMA core thermally decomposes between about 250 and 400 °C, whereas the PAN shell undergoes stabilization and subsequent carbonization under nitrogen at elevated temperatures. As the core polymer decomposes and volatilizes, it leaves behind an internal void while the PAN shell is transformed into a carbon shell. Structural evolution during this process can also lead to shell deformation or partial breakage due to thermal expansion and decomposition of the polymer core [42].

Polymer templates can also be integrated with other precursor systems to generate more complex hollow architectures. For example, polystyrene (PS) spheres coated with a ZIF-67 shell form PS@ZIF-67 composite particles that can be converted into single-holed Co/N-doped HGSs through pyrolysis. During this process, the PS core acts as a thermally degradable template, while the ZIF-67 shell transforms into a nitrogen-doped carbon matrix containing dispersed cobalt nanocrystallites. The large through-hole observed in the final structure originates from the strong hydrocarbon gas outflux generated during thermal decomposition of the PS template, as shown in Figure 3A(II) [43]. Importantly, this example illustrates that shell openings can be deliberately engineered rather than occurring only through stochastic rupture. In such systems, the MOF shell not only preserves the spherical morphology but also serves as a chemically active precursor that introduces heteroatom dopants and catalytic metal species into the carbon framework. These hollow architectures provide accessible internal surfaces and facilitate mass transport through the shell opening. However, the carbon shells produced from polymer-derived precursors are typically thin and structurally delicate. As a result, achieving highly graphitic ordering in these hollow shells may require additional catalytic or structural stabilization strategies, and the resulting carbon frameworks often remain partially graphitized or structurally disordered after carbonization.

In contrast, metal oxide templates such as SiO_2_, MgO, TiO_2_, Al_2_O_3_, Fe_3_O_4_, SnO_2_, Mn_3_O_4_, and CaO expand the thermal processing window and enable higher graphitic ordering [34,35,43,44,45,46,47]. Carbon shells may be formed either by carbonizing coated precursors or by directly depositing sp^2^-hybridized carbon via chemical vapor deposition (CVD), as illustrated in Figure 3B [44,45]. CVD growth often improves shell continuity and electrical conductivity. TEM reveals conformal few-layer graphene coatings, curvature-induced wrinkles, and encapsulated graphitic domains after template removal. These curvature-induced wrinkles may introduce useful strain and defect sites. This means CVD-grown shells are not always perfectly defect-free. Instead, they can naturally form features that allow ions to access the structure. However, removing the template involves some trade-offs. Silica templates require aggressive etching agents such as HF or NaOH. These chemicals increase environmental impact and may also damage the shell structure. MgO templates can be removed under milder conditions. However, shell uniformity may decrease due to particle aggregation if the precursor coating is not carefully controlled [34,43,46,47]. Although oxide templates enhance structural precision and graphitic continuity, template removal often requires aggressive etching that can damage shells and generate chemical waste. Coupled with the inherently multi-step nature of scaffold synthesis, including coating, carbonization, and etching. Coupled with the inherently multi-step nature of scaffold synthesis, coating, carbonization, and etching, these factors introduce cost, energy, and reproducibility penalties that constrain scalable implementation in practical battery systems. Consequently, recent salt-based or water-soluble templating strategies have emerged to prioritize facile dissolution and recyclability over absolute geometric precision, reframing optimization from maximum structural fidelity to maximum process sustainability and industrial compatibility [48].

Metal nanoparticles act as catalytic templates that lower the temperature required for graphitization through carbon dissolution–precipitation or segregation mechanisms, enabling the formation of ordered graphitic shells at substantially reduced processing temperatures. In systems where carbon segregates around metal nanoparticles, subsequent core removal can yield hollow multilayer graphitic structures. The enhanced structural ordering typically improves electronic conductivity, which is beneficial for charge transport in battery electrodes [53]. Catalytic graphitization is inherently size-dependent and dynamic. Larger particles tend to promote the formation of highly crystalline graphite domains (G-effect), whereas smaller particles are more likely to become encapsulated within concentric graphitic shells (Ps-effect), producing carbon onion-like hollow structures. This behavior reflects the balance between carbon–metal interfacial energetics and the thermodynamic driving force for graphitic rearrangement [54]. While increased graphitic ordering improves electrical conductivity, a high degree of structural perfection may reduce structural disorder and defect-associated features that can contribute to ion accessibility, particularly for larger charge carriers such as Na^+^ and K^+^. In addition, encapsulation phenomena associated with the Ps-effect can physically confine metal nanoparticles within robust graphitic shells, which may complicate complete template removal. Therefore, in the design of HGSs for sodium- and potassium-ion batteries, careful control of nanoparticle size and graphitization degree is required to balance conductivity enhancement with preservation of ion-accessible structural features.

Metal–organic framework-derived systems represent a distinct variant in which metal centers act as in situ graphitization catalysts and organic linkers provide both carbon and heteroatom dopants [55,56,57,58]. Upon thermal treatment, the framework decomposes into HGSs whose graphitic order and microchannel structure depend on the nature of the metal node and processing conditions. Iron-based MOFs, for example, are particularly effective in promoting graphitization due to the catalytic activity of dispersed Fe species [55]. Unlike conventional oxide templates, MOF-derived systems enable coupled control over graphitic continuity and dopant chemistry. However, independent tuning of pore architecture, surface area, and chemical composition remains challenging because these features are intrinsically linked to framework decomposition dynamics [56,57,58]. Small changes in heating rate, atmosphere, or precursor infiltration can affect the structure during MOF pyrolysis. These variations may cause structural changes, metal sintering, or pore collapse. This shows that MOF-derived hollow carbons are controlled by linked chemical and structural transformations, rather than only by fixed geometric templating [59,60]. Consequently, MOFs are best viewed as platforms for compositional and microstructural tuning rather than as morphology-driven templates.

Overall, hard-template methods provide the highest geometric precision in controlling cavity size and shell thickness [2,35,36]. However, structural optimization requires careful control of graphitic ordering, porosity, and template removal conditions. Without this balance, precise morphology alone cannot ensure better electrochemical performance. Future progress in HGSs will likely depend less on improving geometric symmetry and more on combining morphology control with tunable microstructure, stable catalytic phases, and environmentally friendly manufacturing methods.

### 3.2. Soft-Template Methods

Soft-template synthesis of HGSs should be viewed not only as a way to control shape but as a framework for influencing structural development. In this process, shell thickness, graphitic ordering, pore structure, and defect chemistry often evolve together during self-assembly and carbonization [50,61,62,63]. Unlike hard-templated systems, soft-template methods do not use a rigid scaffold. In hard templates, the cavity size comes directly from the template, and shell thickness can be adjusted separately. In contrast, soft-template strategies depend on temporary supramolecular structures—such as micelles, block–copolymer aggregates, or emulsified droplets—to generate curvature and confine the precursor [50,63,64]. Because these directing phases decompose during pyrolysis, no post-synthetic etching is required. This can reduce chemical processing intensity and may minimize shell damage associated with aggressive template removal [63,65].

However, the absence of a rigid boundary shifts structural control from geometric inheritance to kinetic synchronization. In surfactant-directed –formaldehyde systems, cooperative assembly between phenolic oligomers and block copolymers (e.g., F127) can produce ordered mesostructures [49,63], which convert into mesoporous carbon shells upon carbonization, as shown in Figure 3C [49]. In typical syntheses, resorcinol and formaldehyde undergo base-catalyzed polymerization in water or alcohol–water mixtures. At the same time, amphiphilic block copolymers such as F127 form micellar aggregates that guide mesoscale ordering. The concentrations of phenolic monomers, the amount of surfactant, catalyst strength, and polymerization temperature all affect this cooperative assembly. These factors ultimately determine the shell thickness and the arrangement of mesopores [49]. After polymerization, the material undergoes controlled thermal treatment. This usually involves carbonization under an inert atmosphere at several hundred degrees Celsius. During this process, the polymer framework converts into conductive mesoporous carbon while partly maintaining the templated structure [49].

Yet cavity formation is not always guaranteed. Hollow interiors typically emerge only when polymerization, phase separation, and micellar organization proceed within a suitable kinetic window; if condensation proceeds too rapidly relative to self-assembly, solid mesoporous spheres or irregular void structures may instead form [63,66]. Thus, shell thickness and cavity evolution are coupled through interfacial energetics and crosslinking kinetics rather than being fully independently programmable variables [50,62].

A mechanistic distinction can arise between micelle-only and emulsion-assisted soft templating. In micelle-directed routes, the mesostructure may exhibit high local ordering, but stabilization of internal voids can depend on differential crosslinking or diffusion-driven depletion of the core region [63,66]. Emulsion–micelle coupling introduces a droplet interface that functions as a mesoscale curvature anchor during early-stage polymerization, enabling more deliberate tuning of cavity size and shell thickness [50]. In these systems, oil–water or solvent–water emulsions are stabilized by surfactants. Carbon precursors then polymerize at the liquid–liquid interface, forming a shell around the droplets. During carbonization, these droplets act as sacrificial cores. The size of the droplets determines the final cavity diameter. This size is controlled by surfactant concentration, stirring intensity, and solvent composition. In contrast, the shell thickness depends mainly on precursor concentration and polymerization time [50]. A representative emulsion-driven co-assembly mechanism illustrating this liquid–liquid interfacial assembly pathway is shown in Figure 3D [50]. Nevertheless, droplet polydispersity and interfacial instability can broaden size distributions, underscoring that structural precision in soft-templated HGSs remains condition-dependent rather than absolute [50,62]. In both regimes, cavity diameter, shell thickness, and pore hierarchy tend to co-evolve and may be difficult to fully decouple without introducing additional structural constraints [50,62].

Thermal treatment further reinforces this coupling. Increasing carbonization temperature enhances sp^2^ continuity and electronic percolation, which are key parameters in hierarchical carbon design, but also promotes shrinkage, aromatization-induced densification, and partial mesopore closure [63,65]. As graphitic ordering increases, defect density and pore accessibility typically decrease, potentially shifting ion transport from open mesopore-dominated pathways toward more diffusion-limited regimes [62,65]. Conversely, amorphous or weakly graphitized carbon frameworks typically exhibit high surface area and abundant defect sites, whereas highly graphitized structures provide improved electrical conductivity and enhanced structural stability [49,65]. In soft-templated HGS, therefore, graphitization degree, shell thickness, and porosity are often interdependent, and thermal processing tends to redistribute these parameters within a coupled structural space [62,63].

From a parameter-driven perspective, soft templating provides synthetic economy and compositional flexibility but generally offers lower geometric determinism compared to hard-template routes [62,64]. Structural metrics including shell uniformity, cavity size distribution, and pore connectivity are therefore governed largely by rate competition rather than rigid replication [50,63]. Consequently, soft-templated HGSs are better evaluated by how effectively they balance graphitic continuity, transport-accessible porosity, and mechanical robustness within this kinetically constrained framework, rather than by hollow morphology alone [50,62].

### 3.3. Self-Templated and Template-Free Approaches

Self-templated strategies generate hollow interiors through intrinsic material evolution rather than through replication of an external sacrificial scaffold. In such systems, hollow structures form through internal physicochemical transformations within the precursor particle rather than by coating and removing a template. Studies on hollow nanostructure synthesis show that several mechanisms can produce this effect, including diffusion imbalance (the Kirkendall effect), galvanic replacement reactions, selective dissolution–reprecipitation, and Ostwald ripening. In all of these processes, material redistributes from the particle interior toward the outer shell during reaction or thermal treatment [67].

Mechanistically, this class spans (i) diffusion imbalance voiding (often framed as Kirkendall-type processes), (ii) selective internal dissolution or conversion driven by compositional or redox gradients, and (iii) heterogeneous contraction during carbonization/thermal treatment that creates a mechanically coherent shell around a depleted core (Figure 3E,F) [51,52,68,69,70]. In diffusion-driven hollowing, different diffusion rates of atoms lead to vacancy accumulation inside the particle, which eventually forms a hollow cavity. In redox-driven processes such as galvanic replacement, a less noble component dissolves, while a more stable phase deposits on the outer surface, gradually creating a hollow structure [67].

These pathways are frequently discussed under the broader “self-templating” umbrella alongside Ostwald ripening-type hollowing, where interior mass is redeposited outward to reduce total interfacial energy. During Ostwald ripening, material from the particle core dissolves because of its higher surface energy and redeposits on the outer shell. Over time, this process enlarges the internal cavity while thickening the surrounding shell [67]. What matters critically, however, is that the shell is not an imposed geometry; it is an emergent outcome of coupled transport, reaction, and densification kinetics. The hollow architecture therefore develops through dynamic redistribution of material rather than replication of a template, which is why self-templating delivers “integrated” shells without a discrete template removal step [67]. Reviews that systematize these categories emphasize that outward diffusion/etching and ripening are not merely different “routes”; rather, they generate distinct defect topologies and shell permeabilities that often govern electrochemical behavior more strongly than the nominal void size [71].

A practical advantage over hard templating is clear: fewer unit operations and reduced chemical waste. Because hollow structures form intrinsically during precursor transformation, self-templated methods avoid sacrificial templates and the harsh chemical etching steps needed to remove them [67]. However, the deeper trade-off concerns where structural control resides. In hard-templated systems, cavity diameter and shell thickness can be independently tuned through template size and coating thickness. In self-templated systems, by contrast, these parameters often co-vary because both are governed by the same rate competitions (precursor decomposition, crosslinking, mass transport, and carbon yield). This coupling is especially pronounced in template-free or quasi-template-free carbon systems. As an early example, Han et al. reported a template-free route to interconnected hollow carbon nanospheres prepared from sucrose by pressure-assisted reduction and graphitization in autoclaves using Zn as a reductant. The resulting materials exhibited hollow spherical morphology, an interconnected structure, and thin graphitic shells with an almost uniform thickness of about 10 nm [72].

By contrast, more recent template-free routes deliberately “program” the chemistry, for example, through polymeric or CTF-like networks and heteroatom-rich precursors, to couple gas evolution with simultaneous pore creation and shell stabilization. Zheng et al. report N/S dual-doped hollow mesoporous carbon nanospheres via a template-free strategy in which heteroatom chemistry stabilizes the mesoporous shell architecture; yet, the same dopant-enabled disorder that improves wettability and kinetics can also increase irreversible adsorption and SEI formation in Na^+^/K^+^ systems if microporosity is not carefully moderated, underscoring that template-free synthesis does not inherently translate into electrochemical efficiency [21].

A representative example of kinetically programmed self-templating is provided by Aslam et al., who engineered a bimetallic ZIF-67/ZIF-8 precursor. During sulfidation, the structure undergoes diffusion-driven phase transformation. This process first forms yolk–shell intermediates and then develops into a single-holed hollow ZnCoS@Co_9_S_8_ structure embedded in N-doped carbon after annealing [73]. In this case, hollow formation did not arise from stochastic gas evolution alone but from diffusion asymmetry, phase transformation (CoS → Co_9_S_8_), and carbon framework contraction acting cooperatively. Crucially, the MOF-derived carbon matrix preserved architectural integrity during mass redistribution, mitigating the shell collapse typically observed in simple Kirkendall-type hollow sulfides. This demonstrates that self-templating can be kinetically engineered through rational precursor design, partially overcoming the geometric indeterminacy often associated with template-free systems. However, structural features such as shell thickness and pore distribution still depend on reaction conditions, including sulfidation and thermal treatment [73].

These approaches also impose a distinctive ceiling (and opportunity) on graphitic ordering. In self-templated carbons, shell graphitization is rarely uniform: local catalytic species (residual metals from MOFs, salts, or intrinsic heteroatoms) and local oxygen/nitrogen functionalities can create spatially heterogeneous ordering, often producing turbostratic carbon with expanded interlayer spacing rather than fully graphitized shells. For Na^+^/K^+^ storage, that disorder and expanded spacing can facilitate insertion and pseudocapacitive uptake; however, expanded spacing and high defect density simultaneously increase surface reactivity and first-cycle irreversible loss. MOF-derived HGSs illustrate this tension particularly well: MOFs offer compositional tunability and in situ metal sites that can catalyze partial graphitization, but their thermal contraction and metal volatilization or redistribution often make shell thickness and pore distribution less predictable than hard-template analogues unless the pyrolysis protocol is engineered to control outward diffusion and collapse [74]. In the same vein, resol–block copolymer deposition strategies described as “self-templating” can yield hollow mesoporous carbon architectures with comparatively more regular mesopore shells than many purely decomposition-driven systems; yet even there, shell formation depends on competitive phase separation and curing kinetics, meaning that apparent regularity is confined to narrow processing windows [75].

In the MOF-derived ZnCoS@Co_9_S_8_/N–C system, moderate graphitic character (as reflected by Raman ID/IG values) indicates partial ordering sufficient for conductivity enhancement, while N-doping improves wettability and charge transport; nevertheless, the large initial irreversible capacity highlights the intrinsic trade-off between defect-enabled kinetics and SEI formation in defect-rich self-templated systems [73].

Self-templated methods improve scalability because they avoid sacrificial templates and harsh etching steps. However, they usually provide less precise control over structural features such as shell uniformity, pore size distribution, and connections between shells compared with hard-templated systems. Better control is possible only when the chemistry is carefully designed to separate the hollowing step from carbonization. This can be achieved through strategies such as staged crosslinking, oxidation before high-temperature treatment, or precursor structures that create internal density gradients. In simple terms, self-templating favors easier processing and chemical integration rather than precise geometric control. The most effective studies treat hollow formation as a process controlled by reaction rates, rather than as a simple by-product of pyrolysis [67].

### 3.4. Biomass-Derived HGS

Biomass-derived carbons represent a chemically versatile route to HGSs in which graphitization, porosity development, and heteroatom incorporation often occur simultaneously during thermal transformation. Unlike conventional template-based approaches, biomass systems do not typically provide independent geometric control over cavity size and shell thickness; instead, hollow formation emerges from coupled chemical and structural evolution. Their relevance to battery applications therefore lies less in morphological novelty and more in their ability to integrate graphitic ordering and defect chemistry within a single processing step.

**Figure 4:**
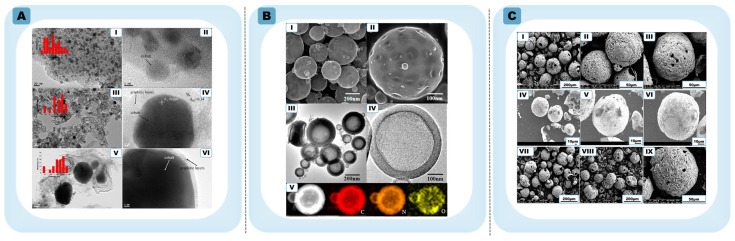
Representative examples of biomass-derived HGSs (**A**) TEM and HRTEM images of Co@C catalysts prepared at different carbonization temperatures: (**I**,**II**) Co@C-400, (**III**,**IV**) Co@C-600, and (**V**,**VI**) Co@C-800. Reprinted with permission from Ref. [78] Copyright 2016 American Chemical Society. (**B**) Morphology and compositional characterization of N-HCSs: (**I**,**II**) SEM images; (**III**,**IV**) TEM images (including high-resolution); (**V**) HAADF-STEM and EELS elemental mapping (C, N, O); Reprinted with permission from Ref. [81] Copyright 2018 American Chemical Society. (**C**) SEM of (**I**–**III**) HCN-400, (**IV**–**VI**) HCN-500 and (**VII**–**IX**) HCN-600.Reprinted with permission from Ref. [82] Copyright 2024 RSC.

Lignin-based systems illustrate this coupling particularly clearly. The introduction of transition metal salts such as Fe during relatively low-temperature carbonization (~600–650 °C) promotes localized catalytic graphitization while simultaneously contributing to internal void formation (Figure 4A) [76,77,78]. In situ-generated metal nanoparticles reorganize carbon into few-layer or multilayer graphitic domains, with interlayer spacing approaching ~0.34 nm, at temperatures significantly lower than those required for conventional graphite synthesis. Subsequent removal of the metal phase generates additional mesoporosity, linking catalytic graphitization directly to pore development. Beyond hollow formation, such systems demonstrate that pyrolysis temperature and metal content govern shell thickness, defect density, and graphitic continuity, thereby influencing electronic transport and ion-accessible surface sites [78]. In this context, biomass-derived HGSs should be viewed as a platform for tuning graphitic order and defect structure rather than merely as examples of catalytic hollowing.

Biomass routes are not limited to metal-assisted processes. Activation-driven approaches, including KOH treatment during aerosol or spray-drying methods, induce internal contraction and mass redistribution that lead to hollow spherical architectures without relying on rigid sacrificial templates [79]. In these systems, activation chemistry simultaneously controls pore generation and cavity evolution. However, increased surface area and mesoporosity are often accompanied by higher defect density and enhanced surface reactivity, which may compromise initial Coulombic efficiency in battery operation. Thus, activation serves as a powerful but non-selective structural lever, improving transport pathways while potentially destabilizing the electrode–electrolyte interface.

Metal-free HGSs can be directly obtained from biomass precursors such as lignin or glucose and glucosamine via a template-free aerosol-assisted self-assembly process, demonstrating that hollow morphology can form independently of catalytic graphitization (Figure 4B) [80,81]. Controlled pyrolysis alone can yield hollow carbons exhibiting measurable graphitic ordering, although typically with greater structural disorder than metal-assisted analogues. Reactive gas treatments, such as CO_2_-assisted carbothermal processes, introduce an additional means of tuning shell continuity, porosity, and residual metal dispersion during thermal evolution (Figure 4C) [82]. These atmospheres influence carbon reorganization and etching dynamics, enabling partial decoupling of graphitic ordering from pore development, albeit within limits imposed by precursor chemistry.

A key advantage of biomass-derived HGSs is the presence of intrinsic heteroatom doping (e.g., N, S, or P) originating from the natural composition of the precursor. Such heteroatoms modify local electronic structure and surface polarity without requiring separate doping steps, directly impacting ion adsorption energetics and interphase formation. Nevertheless, the principal limitation of biomass-based systems lies in feedstock heterogeneity and batch-to-batch variability. Shell thickness, pore size distribution, and graphitization degree are strongly dependent on precursor composition and processing history, complicating quantitative structure–property correlations [83]. Consequently, while biomass-derived HGSs offer sustainability and chemical tunability, achieving reproducible structural metrics comparable to engineered template systems remains challenging.

In summary, biomass-derived approaches are best interpreted as chemically coupled synthesis platforms in which graphitic ordering, defect density, and porosity evolve together. Their strengths lie in integrated doping and moderate-temperature graphitization; their limitations lie in reduced independent control and reproducibility. For battery applications, their effectiveness depends on managing the trade-off between enhanced surface reactivity and stable conductive shell continuity.

A comparative summary of representative HGS synthesis strategies, including their graphitic quality, structural precision, scalability, and typical applications, is provided in Table 1.

## 4. Chemistry-Specific Design Requirements

The structural advantages of HGSs should be considered within the electrochemical context. Their high electronic conductivity, short ion diffusion pathways, and internal void space are generally beneficial. However, the importance of each parameter depends on the battery chemistry. In insertion-based systems, the balance between graphitic order and defect density plays a key role. It influences storage thermodynamics and interphase stability [9,25,31,32]. In metal anode configurations, void architecture and surface energetics regulate nucleation behavior and interfacial evolution [25,28,30,31]. In multivalent chemistries, kinetic mitigation dominates design priorities [29,87,88]. In solid-state batteries, mechanical conformity and chemical compatibility outweigh surface area considerations [33,89,90], whereas in halogen-based systems, adsorption thermodynamics and pore size confinement dictate reversibility [5,91,92,93,94]. Thus, battery performance metrics—including specific capacity, rate capability, Coulombic efficiency, interfacial resistance, and cycling stability—emerge not from universal structural optimization.

**Figure 5:**
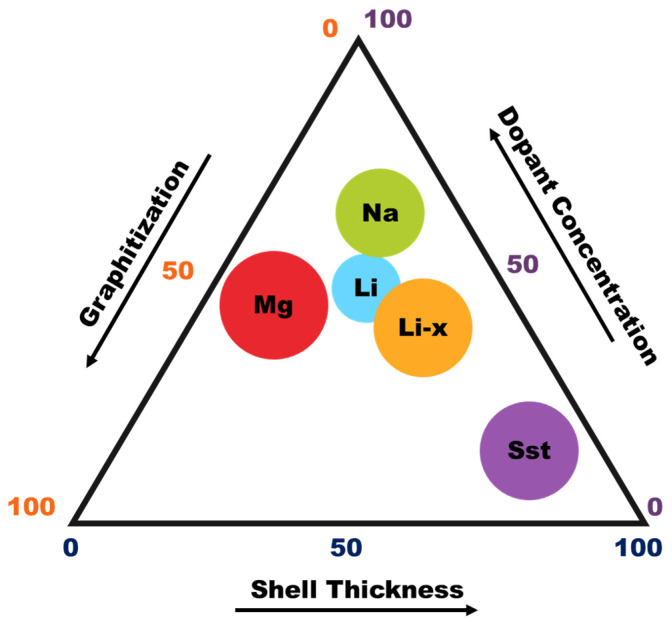
Ternary structural design space of HGS, defined by shell thickness, graphitization degree, and defect density. Each battery chemistry occupies a distinct region within this triangular parameter framework according to its dominant electrochemical constraints.

To unify the structure–chemistry relationships discussed above, HGSs can be conceptualized within a ternary design space defined by shell thickness, graphitization degree, and defect density (Figure 5). These three coupled parameters collectively regulate conductive continuity, mechanical robustness, and surface reactivity [90]. Within this conceptual framework, different battery chemistries may be associated with distinct regions of the triangular space depending on the dominant electrochemical limitations governing their operation, particularly those associated with ion transport kinetics and interfacial charge transfer processes [95]. Importantly, electrochemical optimization does not correspond to a single universal structural extreme; rather, performance typically emerges from a chemistry-dependent balance among these interdependent structural axes.

The positioning of battery chemistries within the ternary parameter space of Figure 5 can be interpreted in terms of their dominant transport and interfacial constraints. In multivalent systems, such as Mg-, Ca-, and Al-based batteries, the high charge density of the working ions leads to stronger Coulombic interactions with host frameworks and generally slower solid-state diffusion than in monovalent systems [96]. Under these conditions, reducing shell thickness can be advantageous because shorter diffusion paths may help alleviate the kinetic penalty associated with ion insertion or interfacial conversion processes. From this perspective, multivalent chemistries can be viewed as trending toward the thin-shell region of the ternary diagram. By contrast, in solid-state systems, the dominant limitation is often the maintenance of continuous ion and electron transport pathways across solid–solid interfaces within the composite electrode [97]. In this case, a higher degree of graphitic continuity can be beneficial, because a well-connected graphitic framework improves electronic percolation and supports stable charge transport pathways during operation [98]. Thus, the locations of different battery classes in Figure 5 should be interpreted as a conceptual representation of whether performance is governed primarily by ion transport distance, interfacial kinetics, electronic continuity, or structural resilience.

### 4.1. Monovalent Ion Batteries (Li^+^, Na^+^, K^+^)

**Figure 6:**
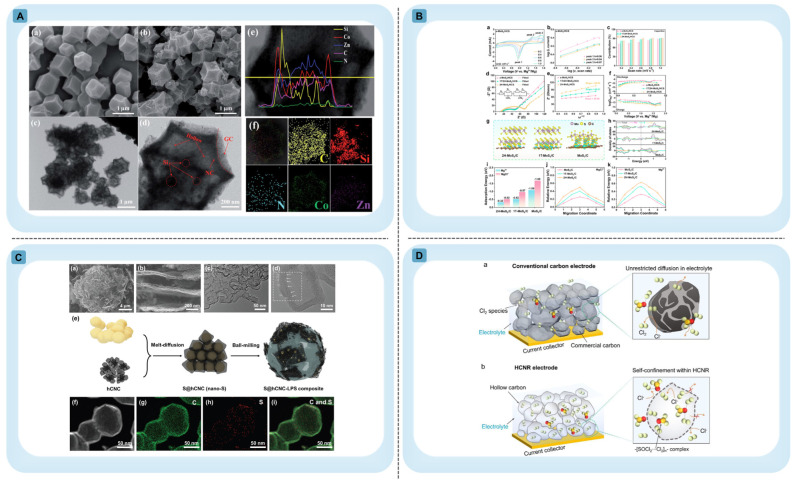
HGSs in different battery systems: (**A**) Structural characterization of Si@ZIF-8@ZIF-67 and Si@H-NC@GC: SEM images (**a**,**b**) and TEM images (**c**,**d**), reveal the morphology of the material, while TEM coupled with EDX line scanning (**e**) and elemental mapping (**f**) confirm the distribution of Si, C, N, Co, and Zn within the structure. Reprinted with permission from Ref. [89] Copyright 2020 RSC. (**B**) Kinetic and theoretical analysis of a-MoS_3_/HCS: (**a**) CV curves recorded at different scan rates; (**b**) determination of b-value from log(i) vs. log(v); (**c**) calculated capacitive contribution ratios; (**d**) Nyquist plots with the fitted equivalent circuit; (**e**) Z′ vs. ω^−1^/^2^ plots; (**f**) calculated Mg^2+^ diffusion coefficients; (**g**) charge density difference maps illustrating electron accumulation and depletion; (**h**) density of states of 2H-MoS_2_/C, 1T-MoS_2_/C, and MoS_3_/C; (**i**) adsorption energies of MgCl^+^ and Mg^2+^; (**j**,**k**) migration energy barriers of MgCl^+^ and Mg^2+^. Reprinted with permission from Ref. [99] Copyright 2024 American Chemical Society. (**C**) Structural and compositional characterization of hCNC and S@hCNC composites: SEM (**a**,**b**) and TEM (**c**,**d**) images of hCNC (arrows indicate broken fringes); (**e**) schematic illustration of S@hCNC fabrication via melt diffusion and cathode assembly; (**f**) STEM image with corresponding elemental mapping of (**g**) C, (**h**) S, and (**i**) combined C and S distribution. Reprinted with permission from Ref. [100] Copyright 2025, the author(s). (**D**) Schematic illustration of the distribution of the -[SOCl_2_···Cl_2_]_n_- complex in Li-Cl_2_ batteries using (**a**) using commercial carbon and (**b**) HCNR cathodes. Reprinted with permission from Ref [101] Copyright 2026 Wiley-VCH GmbH.

In monovalent systems, electrochemical performance is governed by the interplay between insertion into graphitic domains and defect-mediated adsorption [32]. Highly ordered graphite supports Li^+^ intercalation but limits Na^+^ and K^+^ insertion due to their larger ionic radii and unfavorable staging behavior [31]. Conversely, excessive structural disorder enhances adsorption-dominated storage but promotes electrolyte decomposition and heterogeneous solid–electrolyte interphase (SEI) formation [9,25]. Optimal performance therefore arises from moderate graphitization, which preserves electronic percolation while maintaining expanded interlayer spacing and accessible defect sites. Controlled mesoporosity reduces polarization and facilitates electrolyte infiltration [31], whereas excessive microporosity intensifies parasitic reactions. Interphase stability scales with defect density: highly defective shells promote thicker, compositionally heterogeneous SEI layers [9], while moderately ordered domains favor more uniform and mechanically stable interphases with reduced impedance growth [25]. The structural buffering and conductive continuity of hollow double-layer carbon nanocage architectures, as illustrated in Figure 6A [32], exemplify how void space and shell ordering jointly mitigate volume change and maintain electrical percolation. Accordingly, in Li^+^, Na^+^, and K^+^ batteries, structural optimization requires balancing graphitic continuity with controlled disorder rather than maximizing crystallinity.

Within the ternary HGS framework (Figure 4), monovalent systems therefore occupy a regime of moderate shell thickness, intermediate graphitization, and controlled defect density.

### 4.2. Metal Anodes (Li, Na, Zn)

For metal anode systems, the dominant limitation shifts from insertion thermodynamics to nucleation kinetics and current-density distribution [25]. Internal void volume directly regulates effective local current density by redistributing electron flux within a three-dimensional conductive framework, promoting homogeneous plating and suppressing dendritic growth [25,28]. The integrated design logic includes spatial regulation of nucleation, accommodation of volumetric changes, and stabilization of the electrode–electrolyte interface [28,31]. These coupled effects are visualized through top-view and cross-sectional SEM analyses of confined Li deposition within hollow carbon hosts [28]. In this regime, crystallinity is secondary to surface chemistry: lithiophilicity, sodiophilicity, or zincophilicity govern nucleation overpotentials and deposition uniformity more strongly than graphitic order. In aqueous Zn systems, porous graphitic frameworks homogenize Zn^2+^ flux and regulate nucleation behavior, mitigating parasitic reactions [30]. Mechanical robustness of the shell is decisive for dendrite suppression; structurally resilient shells maintain confinement and reduce repetitive interphase rupture during extended cycling [28]. Thus, void architecture, surface energetics, and shell integrity dominate HGS design in metal anode chemistries. Accordingly, metal anode systems shift toward thicker, mechanically robust shells with conductive graphitic continuity and surface-engineered defect chemistry.

### 4.3. Multivalent Systems (Mg, Ca, Al)

Multivalent batteries are intrinsically constrained by strong coulombic interactions and sluggish solid-state diffusion [87]. Under these conditions, HGSs function primarily as conductive reaction scaffolds rather than as direct insertion hosts. Thin shells shorten effective ion transport distances and reduce migration barriers, mitigating kinetic penalties associated with Mg^2+^ diffusion [88]. The interconnected sp^2^ carbon backbone ensures rapid electron transport, compensating for slow ionic mobility within embedded conversion-type active phases. Surface polarity and defect chemistry influence solvation energetics and interfacial charge transfer resistance, making controlled surface engineering more impactful than simply increasing surface area or crystallinity [29,88]. As illustrated in Figure 6B [88], incorporation of hollow carbon scaffolds significantly lowers charge transfer resistance, enhances Mg^2+^ diffusion coefficients, and reduces migration energy barriers. Therefore, in multivalent systems, structural optimization targets kinetic facilitation through thin-shell architectures and tailored interfacial chemistry rather than maximizing storage sites. In the ternary design map (Figure 4), multivalent systems favor thin shells, high graphitic continuity, and moderate polarity defects, while not necessarily requiring high porosity because kinetic limitations are dominated by multivalent transport and interfacial barriers rather than by the number of adsorption sites.

### 4.4. Solid-State Batteries

In solid-state batteries, performance is governed predominantly by the preservation of continuous solid-solid contact among active materials, solid electrolytes, and conductive phases [33]. Mechanical robustness therefore becomes the principal structural requirement. HGSs must withstand stack pressure and conversion-induced volume changes without structural collapse to maintain electronic percolation and extended triple-phase reaction pathways. The hierarchical composite architecture depicted in Figure 6C [33] demonstrates how HGSs sustain intimate contact among sulfur, carbon, and solid electrolyte components. Interfacial chemical compatibility between carbon and solid electrolyte outweighs surface area considerations in determining resistance evolution [89]. Excessively dense graphitic shells may restrict ionic accessibility, whereas excessive microporosity can disrupt continuous ion transport pathways and increase interfacial impedance [89,90]. The macroscopic evolution of solid–solid interfaces, highlighted [90], further illustrates how contact degradation and interphase growth govern long-term impedance and electrochemical stability. Consequently, optimized HGS architectures for solid-state systems require mechanically resilient frameworks with transport-accessible porosity and chemically compatible surfaces. In the ternary design space, solid-state batteries favor high graphitic continuity, low-to-moderate defect density, and structurally resilient shells.

### 4.5. Halogen-Based Systems (Li-Cl_2_)

In halogen-based systems such as Li-Cl_2_ batteries, reversible LiCl/Cl_2_ conversion and gas–solid equilibria define structural requirements [5]. Early demonstrations showed that activated carbon and graphite hosts could physically confine electro-generated Cl_2_ and enable reversible cycling across wide temperature ranges [5]. Subsequent strategies introduced chemical confinement via functionalized MOFs and imine-containing porous nanocages to enhance chlorine adsorption through specific host–guest interactions [91,92], while interhalogen compounds such as ICl_3_ reduced reliance on purely physical trapping mechanisms [93]. More recently, hollow carbon nanoreactors with engineered sub-nanometer micropores have demonstrated size-selective confinement, where mesocavities serve as reaction reservoirs and micropores restrict chlorine escape, as illustrated in Figure 6D [94]. In these systems, adsorption energetics dominate performance, and controlled spatial deposition of LiCl within confined cavities stabilizes interfacial chemistry. Graphitic continuity remains essential to sustain electron transport during repeated conversion cycles, whereas excessive defect density or insufficient confinement compromises reversibility. Hence, precise control of micropore distribution, adsorption chemistry, and conductive integrity defines HGS performance in Li-Cl_2_ batteries.

Across all battery chemistries, HGSs do not exhibit universally optimal structural parameters. Instead, electrochemical performance emerges from the coupled interplay among adsorption processes, insertion pathways, ion transport kinetics, interphase stability, and mechanical resilience. Effective HGS engineering therefore requires chemistry-specific tuning of shell thickness, graphitic ordering, pore architecture, and surface chemistry in response to the dominant electrochemical limitation of each system. However, these particle-level design parameters operate within composite battery electrodes rather than as isolated nanoshells. In practical electrodes, HGS particles assemble into electrode network architectures, forming interconnected networks with conductive additives and polymer binders, where electron percolation, interparticle junction resistance, pore accessibility, and binder distribution collectively determine how effectively intrinsic material properties translate into measurable battery performance. Consequently, the chemistry-dependent structural requirements discussed above must be interpreted within the broader context of electrode-scale network architecture and transport pathways.

### 4.6. Electrode Network Architecture Governs HGS Performance

Most HGS studies implicitly treat nanoshells as isolated performance units, yet practical electrodes operate as composite networks in which transport, reaction uniformity, and mechanical stability are governed by interparticle connectivity and carbon–binder microstructure [102]. Consequently, electrode performance depends less on the intrinsic conductivity of an individual shell than on how shells connect to form continuous electron pathways, how ions access active sites through tortuous pore networks, and how these pathways evolve under densification and cycling [103,104,105]. For HGS, this electrode-scale perspective is not optional: hollow morphology inherently modifies packing, contact mechanics, and binder distribution, so particle-level advantages can be amplified or neutralized depending on network architecture [103].

#### 4.6.1. Percolation and Junction Robustness

Even highly graphitized shells can underperform if shell-to-shell junction resistance dominates [102,103]. Hollow particles often provide limited real contact area, and the conductive benefit of graphitic shells is only realized when contacts percolate through the electrode thickness [102,104,105]. For example, microstructure-resolved modeling of NMC electrodes demonstrates that insufficient conductive network connectivity leads to incomplete utilization of active material and reduced discharge capacity, particularly at high rates where electronic transport limitations become pronounced [105].

In practical electrodes, shell–shell connectivity may arise through discrete point contacts, binder-mediated interfaces, partial graphitic necking formed during thermal treatment, or hybrid bridging via fibrous or sheet-like carbons. However, experimental and modeling studies consistently show that simple point contacts provide high interparticle resistance, whereas continuous conductive networks formed by carbon–binder domains or graphitic bridges significantly reduce potential gradients across the electrode and improve rate capability [105].

Comparative studies of electrode microstructures further demonstrate that insufficient conductive additive content can disrupt this network. For instance, electrodes with excessive active material loading show severe deterioration in high-rate performance due to poor conductive network continuity, resulting in strong potential drops across the electrode and reduced capacity at high C-rates. Conversely, well-connected carbon–binder networks enable efficient electron transport, allowing higher capacity retention and improved utilization of active material during fast discharge [105].

Among these, only percolating necked or bridged architectures provide low-resistance, mechanically resilient pathways; simple physical contact between hollow spheres is frequently insufficient to sustain stable conduction under cycling-induced stress. Recent microstructural analyses also highlight that conductive network must balance electronic connectivity with ionic transport pathways. The carbon-binder domain (CBD), while essential for electron conduction, can simultaneously restrict ionic transport if excessively dense, leading to increased ionic resistance and reduced rate capability in thick electrodes [104]. Manufacturing studies further show that increasing calendaring pressure improves particle contact and decreases interfacial resistance, which can enhance rate performance; however, excessive densification compresses interparticle pores and increases ionic transport resistance, ultimately limiting high-rate capacity [105].

This shifts the design emphasis from “high sp^2^ content” to junction quality and percolation robustness, particularly after calendaring [104]. Over-compression can improve electrical contact but may collapse thin shells or close ion pathways; under-compression preserves porosity but increases contact resistance and exacerbates current localization. Thus, an “optimal” HGS electrode is defined by a coupled window of (i) shell mechanical integrity, (ii) interparticle contact density, and (iii) preserved ionic accessibility, rather than by any single particle metric [103]. Electrodes that achieve this balance exhibit improved active material utilization, higher rate capability, and better cycling stability compared with structures optimized solely for high graphitic shell crystallinity.

#### 4.6.2. Interface Coupling in HGS-Based Composites

In realistic electrodes, HGSs are commonly integrated with secondary phases (Si, metal oxides/sulfides, sulfur, metal plating hosts). Depending on architecture, HGSs may function as (i) a conductive additive dispersed among active particles, (ii) a hollow host encapsulating active phases, or (iii) a continuous carbon scaffold forming the primary electronic backbone. These roles impose different connectivity requirements: additive-type designs demand low percolation thresholds, host-type designs require efficient electron extraction from the cavity to the external network, and scaffold-type designs must maintain structural continuity across the full electrode thickness [102,103]. In all cases, performance is controlled by how effectively the architecture couples three interfaces: (1) active phase–shell, (2) shell–shell, and (3) shell–binder/electrolyte. A frequent cause of failure is that active material utilization becomes limited not by intrinsic reaction kinetics but by incomplete electronic extraction from the encapsulated or anchored phase to the current collector due to discontinuous shell–shell wiring [104,105]. Conversely, when shell connectivity is strong, carbon domains can function as mechanically resilient backbones that preserve electrical continuity during densification and cycling [104].

#### 4.6.3. Carbon Binder Domain Engineered Electrodes

Binder is often treated as a passive additive, yet it directly controls both mechanical cohesion and electrical isolation within composite electrodes [102,104,105]. Excess binder can coat shells and suppress junction conductivity; insufficient binder leads to loss of percolation during cycling [102]. Critically, binder distribution is rarely uniform: drying and processing can generate through-thickness gradients that create electronically well-wired but ion-starved regions (or the reverse). Because HGS morphology affects slurry rheology and packing [103,105], HGS electrodes are particularly susceptible to microstructural drift during processing. Therefore, composite electrode design should explicitly consider binder type, solid loading, mixing protocol, drying conditions, and calendaring pressure as first-order parameters that co-determine shell connectivity and ionic accessibility [104].

#### 4.6.4. The Hollow–Density Trade-Off

A recurrent limitation of hollow architectures is the conflict between gravimetric gains and electrode-level volumetric penalties [103,104]. Increased interparticle void fraction can reduce tortuosity and improve rate capability, but it lowers electrode density and therefore volumetric capacity [105]. Attempting to recover density by stronger calendaring can compromise shell integrity and collapse transport channels. This trade-off is especially important because many HGS reports rely on thin, low-loading electrodes where network and gradient effects are muted [105]. For HGSs to be evaluated under conditions relevant to practical cells, performance should be discussed at meaningful areal capacities and thicknesses, where transport non-uniformity and percolation stability become decisive.

#### 4.6.5. Redefining Performance Descriptors for HGS

To align HGS design with modern electrode expectations, particle-level descriptors (shell thickness, graphitization, and porosity) should be complemented by electrode-level metrics that capture connectivity and architecture, including electrode density (or porosity), thickness/areal loading, and evidence of stable shell–shell percolation after processing and cycling [104,105]. Without this, improvements attributed to “hollowness” remain ambiguous because the dominant limitation may reside at the junction or network level rather than within individual shells [102,103,104].

#### 4.6.6. Recycling and Environmental Impact

The environmental relevance of HGSs in battery electrodes stems from how synthesis pathways, compositional simplicity, and structural durability influence life-cycle burden. Biomass-derived carbon materials are widely recognized as renewable, low-cost electrode candidates that can improve sustainability in energy storage, but their true environmental benefit depends on processing severity; high-temperature graphitization and aggressive activation can negate feedstock advantages if energy use and emissions are not controlled [106]. Carbon frameworks dominated by graphitic sp^2^ domains generally exhibit lower toxicity and greater chemical stability than metal-rich oxides or sulfides, and encapsulating active species within carbon shells can suppress detrimental dissolution, minimizing hazardous release during use and recycling [107]. However, increased compositional complexity or heavy heteroatom loadings can compromise recyclability by complicating separation and increasing processing stages. The choice of templating route is a decisive environmental factor; conventional hard templates requiring hazardous etchants are not compatible with green manufacturing, whereas template-free or MOF-derived hollow carbon syntheses, which avoid sacrificial silica and fluoride etching and reduce chemical waste and processing intensity, making cleaner pathways for hollow carbon formation more accessible [108]. Mechanically resilient carbon backbones in hollow architectures can retain structural integrity after cycling and be repurposed in second-life applications such as capacitive electrodes or catalyst supports without the complex hydrometallurgical treatments needed for traditional metal-rich cathode materials, contributing to circular resource use and reduced waste streams [107]. Ultimately, the environmental advantage of HGSs does not arise automatically from carbon chemistry or hollow morphology but from deliberate control of processing conditions, avoidance of hazardous template chemistries, and design for post-use structural stability that enables recovery and reuse.

#### 4.6.7. Industrial Scale-Up and Life-Cycle Assessment

Life-cycle and industrial production studies show that the sustainability of advanced battery materials strongly depends on energy intensity and supply chain design during large-scale manufacturing. For example, life-cycle assessments of graphite and other battery anode materials indicate that production processes such as graphitization or high-temperature pyrolysis dominate environmental impacts, largely because they require large amounts of electricity. Industrial datasets show that synthetic graphite production can reach carbon footprints of roughly tens of tones of CO_2_ equivalent per ton of material, with electricity consumption alone contributing nearly half of total emissions [109,110]. These results highlight that the environmental feasibility of scaling up battery materials is closely tied to energy source selection, process optimization, and improved manufacturing efficiency, rather than only the intrinsic performance of the materials themselves.

From a broader supply chain perspective, life-cycle analyses comparing conventional mining routes with recycling-based production demonstrate that industrial-scale recycling can significantly reduce environmental burdens, lowering greenhouse gas emissions and resource demand compared with primary extraction [111]. Studies show that recycling lithium-ion batteries into battery-grade materials can reduce environmental impacts, particularly when optimized hydrometallurgical routes and regional energy mixes with lower carbon intensity are used [111]. These findings suggest that sustainable industrial deployment of battery technologies will require integrating circular supply chains, energy-efficient processing, and geographically optimized manufacturing, ensuring that large-scale production remains both economically viable and environmentally responsible.

## 5. Current Limitations and Unresolved Challenges

Despite extensive progress, HGS research remains largely morphology-driven rather than quantitatively engineered. Most studies correlate electrochemical performance with shell thickness, porosity, or defect density qualitatively, yet transferable design metrics linking structural parameters to diffusion length, charge transfer resistance, or interphase growth are rarely established. Recent analyses of hard carbon systems emphasize that predictive structure–performance mapping is still missing in porous carbon anodes, limiting rational optimization and cross-study comparability [3].

A fundamental unresolved issue is precise nanoscale control of shell geometry and uniformity. Variations in shell thickness distribution, incomplete closure, and structural heterogeneity introduce non-uniform diffusion pathways and localized current densities. Of equal importance is the fact that interlinking between nanoshells at the electrode level is rarely engineered deliberately. Poor interparticle connectivity compromises electronic percolation and volumetric packing, meaning that nanoscale optimization does not automatically translate into electrode-scale performance. Hollow shells function within composite electrodes, yet their collective network architecture remains under-addressed.

The well-known trade-off between high surface area and initial Coulombic efficiency (ICE) remains unresolved. Microporosity and defect-rich frameworks enhance adsorption-mediated storage but intensify electrolyte decomposition and unstable SEI formation, directly reducing ICE and long-term stability. Operando investigations confirm that nanopore chemistry rather than total surface area governs parasitic reactions in alkali–ion systems [4]. Thus, maximizing surface area remains a misleading optimization strategy for practical battery electrodes.

Volumetric energy density is similarly under-addressed. Hollow architectures inherently sacrifice packing efficiency, yet most reports emphasize gravimetric metrics without reporting tap density or electrode-level volumetric capacity. Recent discussions on practical anode engineering stress that density penalties frequently outweigh gravimetric gains in porous carbons [6]. Without integrating volumetric constraints, structural optimization remains incomplete.

Doping strategies introduce additional complexity. While heteroatom incorporation enhances surface reactivity and ion affinity, long-term dopant stability, migration, and structural evolution under cycling remain insufficiently understood. Excessive defect density can disrupt conductive continuity and accelerate interphase instability. Moreover, life-cycle assessment (LCA) data for doped and templated hollow carbons are limited, and sustainability claims are often qualitative rather than quantitatively benchmarked against synthetic graphite or alternative carbons. Renewable feedstock alone does not guarantee reduced environmental burden.

Energy intensity represents another structural paradox. Higher graphitic order improves conductivity and structural resilience, yet high-temperature graphitization, CVD growth, and reduction treatments substantially increase embodied energy and processing cost. Recent industrialization analyses emphasize that process complexity and chemical intensity ultimately determine scalability [112]. Without reducing thermal and chemical processing demands, HGSs risk remaining academically optimized but industrially constrained.

Collectively, these challenges reveal a central limitation: HGSs are often optimized at the particle scale, while transport uniformity, interlinking, volumetric efficiency, dopant stability, and manufacturing intensity are insufficiently integrated into a unified design framework.

## 6. Outlook: Toward Parameter-Driven Engineering

Future progress requires shifting from morphology-driven exploration to parameter-driven engineering. Quantitative mapping between shell thickness, pore architecture, and ion diffusion kinetics must replace qualitative “thin vs. thick” comparisons. Emerging porosity-engineering frameworks demonstrate how structural parameters can be translated into measurable transport metrics, providing a pathway toward predictive optimization [113].

Artificial intelligence and machine learning approaches offer a promising route to accelerate predictive design, enabling high-throughput screening of shell thickness distributions, defect densities, and dopant configurations against transport and stability constraints. Integrating experimental datasets with data-driven models could transform hollow carbon development from empirical iteration to predictive optimization.

Operando and in situ methods must also become central rather than supplementary. Real-time tracking of interphase evolution, shell deformation, and confined metal deposition will clarify failure mechanisms that ex situ characterization cannot resolve [4]. Such insights are essential for linking structural design to durability. Coupled electro–chemo–mechanical modeling should be expanded to explicitly include interparticle interlinking and electrode-level architecture, ensuring that nanoscale shell optimization translates into macroscopic performance. HGSs redistribute stress, ion flux, and electronic pathways simultaneously; predictive models must therefore integrate mechanics, transport, and network connectivity. Equally critical is standardized reporting. Beyond surface area and gravimetric capacity, consistent disclosure of tap density, volumetric capacity, initial Coulombic efficiency, pore size distribution, and quantitative defect metrics is necessary to enable meaningful cross-study comparison. Recent hard carbon design reviews emphasize that industrial relevance depends on such comparable datasets [3].

Finally, integration into circular electrochemical systems should guide future materials design. HGSs must be engineered not only for performance but also for recyclability, reduced chemical intensity, and compatibility with electrode-level recovery strategies. Designing shells with structural durability, controlled dopant chemistry, and simplified compositions will support reuse and second-life deployment within sustainable battery ecosystems.

Future progress depends on transforming HGSs from morphology-driven concepts into quantitatively engineered battery components constrained by transport uniformity, interfacial stability, volumetric efficiency, network interlinking, and life-cycle responsibility.

## Figures and Tables

**Table 1:** Representative Strategies for the Synthesis of HGS.

Synthesis Method	Precursor	Template	Graphitic Quality	Structural Precision	Scalability	Application	Ref.
Hard-template	Ethanol	Al_2_O_3_, TiO_2_, MgO SiO_2_	Good graphitic structure	Core–shell nanostructures with uniform graphene coating	High	Electrochemical energy storage (battery electrodes)	[34]
Hard-template	Benzene	In situ formed MgO	Few-layer graphitic graphene shells with high sp^2^ carbon	Uniform graphene coating around nanoparticles,	High	Li-S Battery	[35]
Hard-template	Ferrocene	SiO_2_	High-quality graphitic graphene layers	High structural control over few-layer graphene sheets	High	Sensors, nanoelectronics, energy devices	[84]
Hard-template	PAN	PMMA	mostly amorphous/partially graphitized carbon	Uniform hollow carbon nanospheres	Moderate	Catalytic supports/energy storage	[42]
Hard-template	ZIF-67 (2-methylimidazole-based MOF)	PS	Nitrogen-doped graphitic carbon framework formed after carbonization	Single-holed hollow Co–N–C nanostructure with controlled morphology	Moderate	Oxidase-mimicking nanozyme for chemiluminescent biosensing of β-galactosidase	[85]
Soft-template	Resorcinol + Formaldehyde	Pluronic surfactant micelles	Low–moderate	Highly uniform	Moderate	Li–S batteries	[63]
Soft-template	Dopamine	oil–water emulsion droplets	Low–moderate	Highly uniform	Moderate	Energy storage, catalysis, adsorption	[65]
Soft-template	Coal tar pitch	no external template	High graphitic quality	Nanoporous carbon with graphene walls but less precise morphology control	High	Energy storage (supercapacitors), adsorption	[66]
Soft-template	2,4-Dihydroxybenzoic acid	P123/SO emulsion (PEG-assisted)	Mostly amorphous	controllable shell structures	Moderate	Dye adsorption	[86]
Self-template	Phenolic resin precursors	Silica-like Stöber-derived spheres	Moderate graphitic carbon after heat treatment	Highly uniform hollow spheres	High	Adsorption, catalysis, energy storage	[51]
Self-template	ZIF-8 MOF + Mo (CO)_6_	ZIF-8 framework as sacrificial template	ZIF-8 framework as sacrificial template	Precise nanocage morphology preserved during pyrolysis	Moderate	Heterogeneous catalysis	[52]
Self-template	Metal–organic frameworks	Intrinsic MOF porous framework	Moderate graphitic ordering depending on pyrolysis temperature	Good structural retention of porous framework	Moderate	Energy storage and electrocatalysis	[70]
Self-template	Zn–Co MOF precursors	MOF-derived hollow core–shell template	Conductive N-doped carbon matrix	High control of single-hole hollow core–shell structure	Moderate	Lithium-ion battery electrode materials	[73]
Self-template	Metal–organic frameworks	MOF	Moderate–high graphitic carbon after pyrolysis	Good control of hollow architecture and mesoporous shells	Moderate	Energy storage, catalysis, adsorption	[75]

## Data Availability

No new data were created or analysed in this study. Data sharing is not applicable to this article.
